# The associations of early specialization, sports volume, and maturity status with musculoskeletal injury in elite youth football players

**DOI:** 10.3389/fphys.2023.1183204

**Published:** 2023-05-11

**Authors:** Xiang Li, Runze Feng, Shiyi Luo, Chunman Li, Miguel A. Gómez-Ruano

**Affiliations:** ^1^ China Football College, Beijing Sport University, Beijing, China; ^2^ Faculty of Physical Activity and Sport Sciences, Universidad Politécnica de Madrid, Madrid, Spain

**Keywords:** youth development, athletic injuries, school football, sports participation, student-athletes

## Abstract

**Background:** Youth football in schools has experienced rapid growth in China. Despite the increase of players engaging in more frequent, intensive, and organized sports training at their early ages, the controversy over early specialization (ES) still exists. This study aims to: a) investigate the training situation of players in the Chinese School Football Programme and b) examine the associations of early specialization, sports volume, and maturity status with musculoskeletal injury.

**Methods:** A cross-sectional survey was used. Players who participated in the National School Football Winter Camp were invited to fill out a questionnaire that included the data of maturity, ES, sports volume, and injury history (*n* = 88 boys and *n* = 90 girls).

**Results:** The results have shown that 80.3% of the athletes were classified as ES, while 19.7% of them were classified as non-ES. Almost all athletes (96%) participated in a sport for more than 8 months in a year. Most athletes (75.8%) spent more than twice of the time on organized sports than leisure activities. 30.3% of the athletes trained on average more hours per week than the number of their ages. Binomial logistic regression models reflected the significant differences in the odds ratios (OR) of reporting a history of injury among athletes with different levels of specialization (*p* = 0.024) and the OR of reporting a history of leg injury among players with different weekly sports volumes (*p* = 0.038). Significant differences were also shown in the OR of players reporting foot injuries between players with different maturity states (*p* = 0.046), and the Chi-squared test showed significant differences in the OR of reporting acute injuries between players with different levels of specialization (*p* = 0.048) and weekly activity (*p* = 0.022). No significant differences were found between the remaining variables.

**Conclusion:** Most school football elite players follow the ES pathway even though ES increases the risk of injury, especially acute injury. Pre-pubertal and early pubertal players have a higher incidence of foot injuries. Players who train more hours per week than their ages have more leg injuries and acute injuries. Therefore, priority protection and intervention should be carried out for populations with a high risk of injury.

## 1 Introduction

China has always been committed to the development of football, but it has not been smooth sailing. As reported, from 1995 to 2007, the number of teenagers participating in football training in China dropped sharply from 650,000 to 30,000 (Wang and Gao, 2009). To solve the problem of the continuous decline in the number of young football players, in 2009, the Ministry of Education of China and the Chinese Football Association jointly launched the School Football activity (Ministry of Education of the People’s Republic of China and Chinese Football Association, 2009), in 2015, the General Administration of Sport of China and the Ministry of Education of China jointly enacted a School Football Programme (SFP) (General Office of the State Council of the People’s Republic of China, 2015). The purpose of these initiatives is to increase the population of football, cultivate elite reserve football talent, and explore a new pathway for cultivation by introducing football into extracurricular activities.

Chinese football youth training had always followed a traditional model of “three togethers”, that is, players are centralized in the football school or professional academy to train together, study together, and live together on the campus, which results in the detachment of young players from social environment (Li and Zhang, 2012). This largely hinders the development of adolescent psychology, academics, social skills, and normal identity development (Myer et al., 2015; Popkin et al., 2019). Moreover, there exists a serious problem of early specialization among young players, that is, from the time they joined the professional academy, they stopped playing all other sports and only received high-intensity football training throughout the year (Wang and Gao, 2009). In order to solve the problems mentioned above, a new pathway was initiated as SFP adopted the “three returns” model instead of “three togethers”, allowing young players to return to their families, return to schools, and return to society and improve students’ physical health, promote football, and cultivate reserve football talents by launching football leagues and related activities at school (General Office of the State Council of the People’s Republic of China, 2015; Ministry of Education of the People’s Republic of China, 2019).

According to the latest data from the Department of Sports, Health, and Arts of the Ministry of Education of China, there are currently more than 30,000 SFP-featured schools in China, and 55 million students participate in SFP training and competitions (Ministry of Education of the People’s Republic of China, 2019). Among them, primary school and junior high school students are the main populations. It seems that the SFP has effectively expanded China’s football population and strengthened the foundation for the development of Chinese football. Meanwhile, this programme has made progress in popularizing football and further carries out elite training based on popularization, which is, selecting youth players in school football for elite training (Ministry of Education of the People’s Republic of China, 2019). In 2017, four “Man Tian Xing” (“Starry Sky”) elite youth football training academies were established throughout the country, with the objective of elite training for youth players with potential talents. Until 2019, 80 SFP “Man Tian Xing” academies have been established across China, turning into an important pilot project to promote the improvement of school football competition and the development of youth football players (Zhao et al., 2020). In 2015, the Ministry of Education of China held the first national youth school football summer camp with teams assembled by each province in China and selected outstanding players among them to establish a national team to compete in international competitions.

However, elite youth football players with more potential may experience more stress than recreational football players. This pressure may stem from their internal pressures, for example, players’ inherent desire to excel and their desire to be recognized as talented by coaches, social media, the sports industry, and society (Malina, 2010; Mostafavifar et al., 2013). This pressure can also be extrinsic, such as parental and coaching support, in the hope that young players can gain competitive advantages at an early age (Feeley et al., 2016; Padaki et al., 2017); the incentive of potential college scholarships and future career contracts with lucrative financial rewards (Malina, 2010; Mostafavifar et al., 2013; Padaki et al., 2017); and pressures for early talent identification and selection driven by national elite player development programs (LaPrade et al., 2016; Sugimoto et al., 2017). This stressful environment has resulted in an increasing number of young players choosing to specialize at an early age, with preteens, seventh graders, and/or children younger than 12 years participating in intensive training in organized sports on an annual or approximately year-round basis and/or competed for more than 8 months and played only one sport and no other or limited free play (LaPrade et al., 2016). The early specialization pathway has always been respected in China, especially influenced by the success of Eastern European communist countries in the Olympic Games (Malina, 2010), the coaches inherited its “San Cong Yi Da” training principle, that is, following rigorous, difficult, and realistic high-intensity training will cultivate future champions. The early specialization pathway helped China produce many Olympic champions, especially in individual sports. In soccer, too, there have been many studies showing the importance of extensive soccer-specific practice for reaching elite levels (Ford et al., 2009; Ford et al., 2012). In addition, the “10-year rule” (Simon and Chase, 1973), the theory of “deliberate training” (Ericsson et al., 1993), and later the “10,000-h rule” (Hodges, 1995) all supported athletes to engage in early specialized training.

However, the debate over early specialization has long been a hot topic in the field of youth athlete development. Many researchers believe that early specialization may be detrimental to the long-term development of adolescent athletes, and it is associated with burnout and dropout, while also increasing the risk of overuse injury (Mostafavifar et al., 2013; Brenner et al., 2016; LaPrade et al., 2016). For example, previous research found that sports specialization affects lower body coordination in adolescent athletes, resulting in athletes with less stable hip and knee joints and more likely to get injured (DiCesare et al., 2019). It has also been claimed that the maturity of youth athletes in the specialization pathway affects the risk of injury (Le Gall et al., 2007).

For these aforementioned reasons, some researchers also proposed alternative pathways of early specialization, namely, the early diversification pathway and early engagement pathway (Baker, 2003; Côté et al., 2007; Ford et al., 2009). The early diversification pathway means that athletes engage in not only the primary sport but also other different sports activities during childhood with late or delayed specialization. The early engagement pathway refers to athletes engaging in high amounts of playing form activities in the primary sport, rather than in multiple other sports. However, there is a debate on whether early specialization is detrimental to athlete development or not. Recent studies showed different findings. For example, McGowan et al. (2020) mentioned that early sport specialization did not increase the odds of reporting an injury history; Rugg et al. (2021) surveyed athletes across multiple sports and concluded that early specialization had no effect on scholarship, and specialized athletes have similar career lengths as the athletes who did not specialize; Similarly, Meisel et al. (2022) did not explore any significant relationship between early specialization and basketball-related injuries or mental or physical exhaustion either. Furthermore, Ford et al. (2012) surveyed 328 elite youth football players from different countries and suggested that most of them followed a mixture of the early engagement and specialization pathways; Zibung and Conzelmann (2013) surveyed 159 former Swiss football talents, players who did a great amount of specialized training at an early age were shown to be more likely to achieve high levels of football performance at their peak performance age; Likewise, Sieghartsleitner et al. (2018) surveyed 294 youth players who entered a Swiss football talent development program and found that a large number of specific learning activities increase a player’s chances of achieving great success later in his football career, such as entering a junior national team.

Given that different development pathways may lead to different outcomes in different contexts, country- or cultural-specific contexts may also lead to inconsistencies in outcomes (Baker et al., 2003). For example, the research of Ford et al. (2012) found that the development pathways of adolescents in different countries are not the same. In addition, many factors affect the development of young athletes. It not only depends on the players’ technical and tactical level (Ali, 2011) but also depends on the players’ physiological characteristics (Stølen et al., 2005) and psychological characteristics (Van Yperen, 2009). As mentioned above, the results of the relationship between early specialization and the risk of injury are not the same in the different national backgrounds. At present, there is no relevant research in China to investigate the early specialization of football players and the risk of injury. The background of relevant research is mostly concentrated in Europe, North America, and other regions, and there is a lack of sample research in Asia. At the same time, the growth and development characteristics of adolescents in European and American countries are different from those in Asian countries, and the responses to early specialization may be different. Therefore, this study aims to 1) investigate the training situation of Chinese elite youth football players under the new youth football player development model (SFP), exploring the category to which this model belongs; and 2) examine the associations of early specialization, sports volume, and maturity status with musculoskeletal injury in Chinese elite youth football players. This study is expected to be the first one to investigate the early specialization of Chinese youth football players.

## 2 Methods

### 2.1 Sample

In this study, samples were recruited in the 7-day National School Football winter Camp. All the players who partook in this winter camp were invited to participate in this research. A total of 178 elite youth football players (age = 13.3 ± 1.2 years; *n* = 88 boys and *n* = 90 girls) accepted to fill out face-to-face paper questionnaires about their sports participation and injury history in the past 12 months. Participants in the winter camp first went through the selection of provincial and municipal school football camps to form a representative team of each age group, and then represented the province and the city to participate in the national school football summer camp. During the summer camp, through two rounds of selection, the eligibility for the winter camp is finally determined. After a week of evaluation of training and competition, the players participating in the winter camp will be selected to form the SFP national teams of all ages to participate in international competitions. The total number of players participating in this winter camp is *n* = 193. To ensure a sufficient sample size to represent all players participating in the national school football winter camp, an online sample size calculator was used (https://www.calculator.net/sample-size-calculator.html) (Denscombe, 2010). Assuming a 95% confidence level and a 3% margin of error, a sample size of at least *n* = 164 out of *n* = 193 people was required. Ethical approval was granted by the institution associated with the study on 15 December 2020, reference number BSUCFCIRB-10043202005.

### 2.2 Design and procedure

During the winter camp, a team of seven research assistants and seven team physicians (or physiotherapists) collected data. Researchers invite participants who fit the target population to fill out a questionnaire. The participants voluntarily completed the survey, and all of them were asked for the consent of their parents and coaches before filling out the questionnaire. Some information in the questionnaire must be provided by the players’ parents. Therefore, before filling out the questionnaire, the research assistant and the coaches contacted their parents by WeChat or phone call to ensure the accuracy of the information related to parents in the questionnaire.

### 2.3 Measurements

**FIGURE 1:**
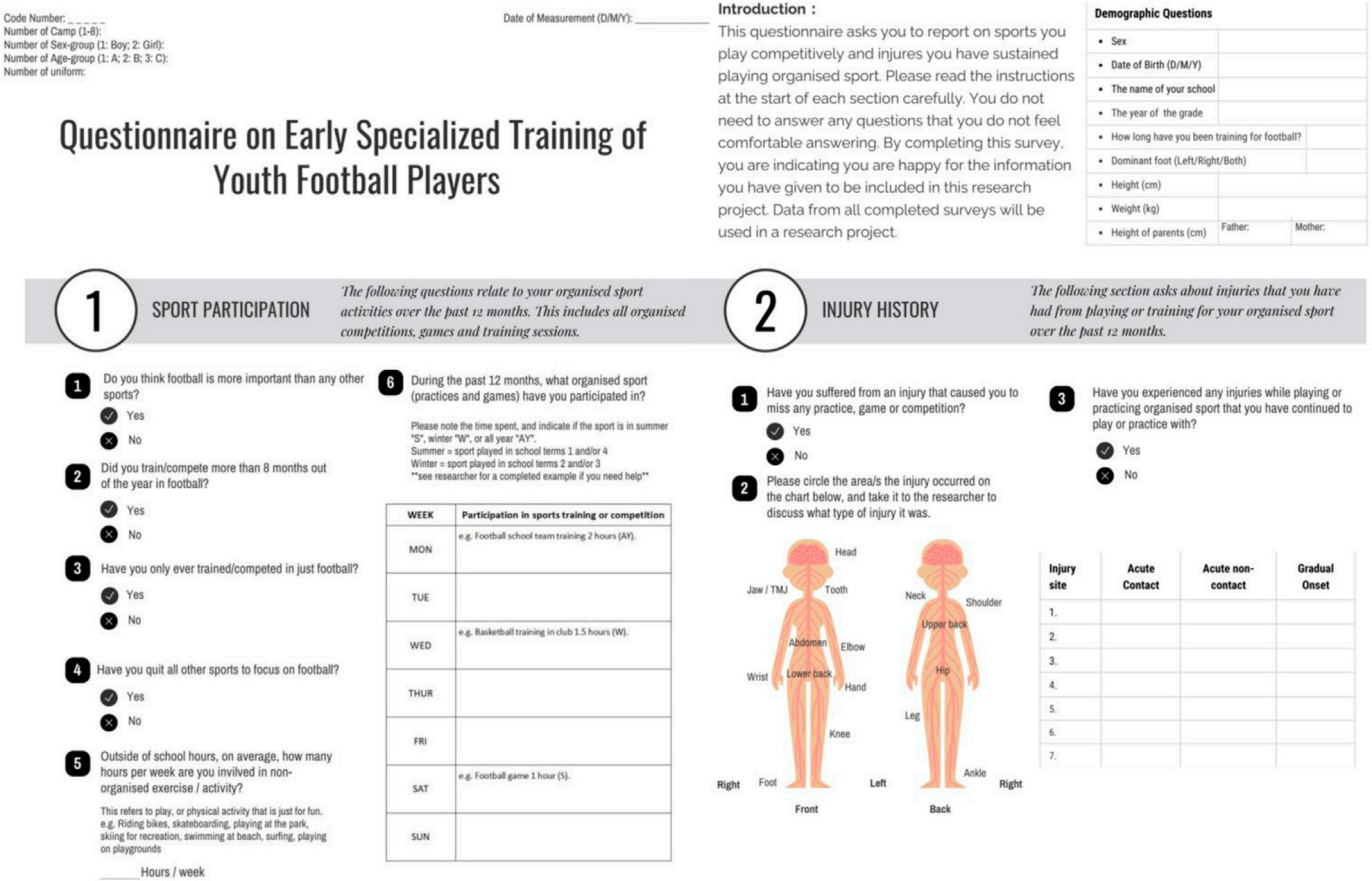
Questionnaire on Early Specialized Training of Youth Football Players (English version).

The questionnaire for this study was adapted from previous study in the relevant field (see Figure 1) (McGowan et al., 2020). The questionnaire contains three sections: demographic data (containing a simple measure of athlete maturity), sports participation, and sports injury history.

#### 2.3.1 Measurement for ES

To assess the level of specialization of an athlete, the questionnaire includes four questions to be answered “yes” or “no” (see Figure 1. Q1.1-1.4). One point was awarded for each “yes” answer, with athletes scoring greater than or equal to 3 being rated as “highly specialized”, those scoring 2 as “moderately specialized” and those athletes with a score of 1 or 0 were rated as “lowly specialized”. As all participants in this study were below the seventh-grade (LaPrade et al., 2016), players rated as “highly specialized” were classified as “early specialized” for this study.

#### 2.3.2 Measurement for maturity

To assess the maturity of the players, players’ gender, date of birth, height, weight, and parental height were collected from the demographic data of the questionnaire to calculate the percentage of a player’s predicted adult height as an alternative indicator for maturity status (Khamis and Roche, 1994). The height of each player’s biological parents was self-reported, therefore, this study used equations from Epstein et al. (1995) to adjust for possible parental overestimation. Early Pubertal was defined as between 85% and 90% of predicted adult height (PAH), and Mid-Pubertal was defined as between 90% and 95% of PAH. Subsequently, Pre-Pubertal was < 85% and Late Pubertal was >95%.

#### 2.3.3 Measurement for sports participation and injury

Sports participation which includes weekly participation in sports training, competitions and leisure free play was recorded in Q1.5-1.6. To assess athletes’ sports injuries over the past 12 months, each athlete who responded with a history of sports injuries was checked by a research assistant and a physician to confirm the location and type of the injury. An acute injury was defined as an injury resulting from a sudden, specific, identifiable event, and a gradual onset injury was defined as any physical complaint not resulting from a suddenly single, identifiable event, but from repeated microtrauma, and could include growth-related pain and overuse issues (Fuller et al., 2006). Others have included the term “overuse injury” in the definition of “gradual onset”. All injuries were recorded regardless of whether time was lost from sport or not.

### 2.4 Data analysis

The data were first input using an Excel spreadsheet for descriptive statistical analysis. All statistical data analyses were completed using the Statistical Programme for Social Sciences IBM SPSS for Macintosh (IBM Corp., Armonk, NY), version 29. Univariate analyses were conducted for the outcome variable (injury reported or not) in seven categories: “any injury”, “gradual onset injury”, “acute injury”, “leg injury”, “knee injury”, “ankle injury” and “foot injury”. The independent variables investigated were level of specialization (low/moderate or high), annual sports volume (whether the player played one sport for more than 8 months of the year, or not), weekly sports volume (whether the player participates in more organized sport per week in hours than their age in years, or not), the ratio of training and leisure (whether the player participates in more than twice the amount of organized sport to recreational free-play activity each week, or not), and maturity status (Pre/Early Pubertal, or Mid/Late Pubertal). Gender (male or female) as a potential confounding variable was controlled for in the logistic regression equation if it was significantly associated with injury in an independent chi-square analysis. To investigate the associations with injury, Pearson’s Chi-squared test and Fisher’s exact test were initially conducted. A *p*-value of < 0.05 was considered a statistically significant association. Phi, which is a chi-square-based measure of association, was calculated to interpret the effect size where 0.1 represents a small effect, 0.3 represents a medium effect, and 0.5 represents a large effect. Binomial logistic regression models were then built to examine the effect of the level of specialization (early specialization or not), sports volume (exceeding each recommendation or not), and maturity status (Pre/Early Pubertal, or Mid/Late Pubertal) on each injury outcome. Odds ratios (OR) with 95% confidence intervals (CI) were calculated.

## 3 Results

### 3.1 Early specialization and sports volume

80.3% of the athletes (*n* = 143) were classified as early specialization (highly specialized), 19.7% of the athletes (*n* = 35) were classified as not early specialization, which includes 16.9% of the athletes (*n* = 30) were moderately specialized, and 2.8% of athletes (*n* = 5) were lowly specialized. Almost all athletes (*n* = 172) participated in a sport for more than 8 months in a year. Most athletes (*n* = 135) spent more than twice of the time on organized sports than leisure activities. In addition, 30.3% of the athletes (*n* = 54) trained on average more hours per week than the number of their ages.

#### 3.1.1 Maturity

Regarding the maturity status of athletes, 20.8% of athletes (*n* = 37) were classified as Pre-Pubertal, 23.0% of athletes (*n* = 41) were classified as Early Pubertal, 39.9% of athletes (*n* = 71) were classified as Mid-Pubertal and 16.3% of athletes (*n* = 29) were classified as Late Pubertal.

### 3.2 Injury

All participants reported a total of 370 unique sport-related injuries over the past 12 months. Of these, 81.3% were acute injuries (*n* = 301) and 18.7% were gradual onset injuries (*n* = 69). Overall, the majority of all reported injuries were lower limbs (71.6%, *n* = 265), and a few were upper limbs (16.2%, *n* = 60) or torso and head (12.2%, *n* = 45). Please refer to the supplementary documents for specific injury distribution.

### 3.3 Univariate analysis

The results of statistical analysis showed that there were significant differences in the odds ratios (OR) of reporting a history of injury among athletes with different levels of specialization (see Table 1); there were significant differences in the OR of reporting a history of leg injury among players with different weekly sports volumes (see Table 2); The OR of players reporting foot injuries were significantly different between players with different maturity state (see Table 3); The OR of reporting acute injuries were significantly different between players with different levels of specialization and weekly activity (see Table 4). The binomial logistic regression was not statistically significant (*p* > 0.05) between the remaining dependent variable (knee injury, ankle injury, and gradual onset injury) and the independent variables (specialization, sports volume, and maturity) (See Tables 5–7).

**TABLE 1:** The association of any injury with specialization, sports volume, and maturity.

Independent variable		Odds ratio (95% confidence interval)	*p*	χ 2	*p*	Phi
Level of Specialization	No ES	0.326 (0.124–0.860)	0.024*	4.309	0.038*	0.156
	ES	3.066 (1.162–8.090)				
Week Sport Volume	≤Age	0.435 (0.134–1.413)	0.166	3.221	0.073	0.135
	>Age	2.301 (0.708–7.484)				
Annual Sport Volume	≤8 months	762,295,468.7 (0.000-)	0.999	1.062	0.596	0.077
	>8 months	0.000				
Training Leisure Ratio	≤2:1	1.008 (0.365–2.788)	0.987	0.127	0.721	0.027
	>2:1	0.992 (0.359–2.742)				
Maturity	Pre/Early Pubertal	1.187 (0.468–3.009)	0.717	0.028	0.866	0.013
	Mid/Late Pubertal	0.842 (0.332–2.135)				

**TABLE 2:** The association of leg injury with specialization, sports volume, and maturity.

Independent variable		Odds ratio (95% confidence interval)	*p*	χ 2	*p*	Phi
Level of Specialization	No ES	1.113 (0.481–2.577)	0.802	0.007	0.932	0.006
	ES	0.898 (0.388–2.080)				
Week Sport Volume	≤Age	0.488 (0.248–0.962)	0.038*	4.524	0.033*	0.159
	>Age	2.047 (1.040–4.031)				
Annual Sport Volume	≤8 months	0.797 (0.121–5.269)	0.814	0.027	0.869	0.012
	>8 months	1.254 (0.190–8.289)				
Training Leisure Ratio	≤2:1	0.775 (0.363–1.653)	0.509	0.966	0.326	0.074
	>2:1	1.291 (0.605–2.754)				
Maturity	Pre/Early Pubertal	0.860 (0.452–1.637)	0.646	0.023	0.880	0.011
	Mid/Late Pubertal	1.163 (0.611–2.214)				

**TABLE 3:** The association of foot injury with specialization, sports volume and maturity.

Independent variable		Odds ratio (95% confidence interval)	*p*	χ 2	*p*	Phi
Level of Specialization	No ES	0.339 (0.074–1.549)	0.163	0.915	0.339	0.072
	ES	2.946 (0.646–13.444)				
Week Sport Volume	≤Age	0.580 (0.248–1.359)	0.210	2.884	0.089	0.127
	>Age	1.723 (0.736–4.034)				
Annual Sport Volume	≤8 months	6.298 (0.636–62.317)	0.116	1.203	0.267	0.082
	>8 months	0.159 (0.016–1.571)				
Training Leisure Ratio	≤2:1	1.315 (0.523–3.302)	0.560	0.672	0.412	0.061
	>2:1	0.761 (0.303–1.911)				
Maturity	Pre/Early Pubertal	2.356 (1.015–5.471)	0.046*	5.581	0.018*	0.177
	Mid/Late Pubertal	0.424 (0.183–0.985)				

**TABLE 4:** The association of acute injury with specialization, sports volume, and maturity.

Independent variable		Odds ratio (95% confidence interval)	*p*	χ 2	*p*	Phi
Level of Specialization	No ES	0.417 (0.171–1.015)	0.054	3.900	0.048*	0.148
	ES	2.398 (0.985–5.839)				
Week Sport Volume	≤Age	0.392 (0.147–1.042)	0.061	5.283	0.022*	0.172
	>Age	2.554 (0.959–6.797)				
Annual Sport Volume	≤8 months	3.122 (0.305–31.934)	0.337	0.100	0.752	0.024
	>8 months	0.320 (0.031-3.276)				
Training Leisure Ratio	≤2:1	1.097 (0.456–2.636)	0.837	0.060	0.806	0.018
	>2:1	0.912 (0379–2.192)				
Maturity	Pre/Early Pubertal	1.209 (0.547–2.671)	0.639	0.158	0.691	0.030
	Mid/Late Pubertal	0.827 (0.374–1.827)				

**TABLE 5:** The association of knee injury with specialization, sports volume, and maturity.

Independent variable		Odds ratio (95% confidence interval)	*p*	χ 2	*p*	Phi
Level of Specialization	No ES	0.809 (0.353–1.855)	0.617	0.041	0.839	0.015
	ES	1.236 (0.539–2.833)				
Week Sport Volume	≤Age	0.600 (0.307–1.172)	0.135	1.752	0.186	0.099
	>Age	1.668 (0.853–3.260)				
Annual Sport Volume	≤8 months	3.020 (0.462–19.742)	0.249	1.249	0.408	0.084
	>8 months	0.331 (0.051–2.165)				
Training Leisure Ratio	≤2:1	1.537 (0.754–3.130)	0.237	1.056	0.304	0.077
	>2:1	0.651 (0.320–1.325)				
Maturity	Pre/Early Pubertal	0.825 (0.443–1.538)	0.545	0.035	0.851	0.014
	Mid/Late Pubertal	1.212 (0.650–2.258)				

**TABLE 6:** The association of ankle injury with specialization, sports volume, and maturity.

Independent variable		Odds ratio (95% confidence interval)	*p*	χ 2	*p*	Phi
Level of Specialization	No ES	0.843 (0.367–1.938)	0.688	1.374	0.241	0.088
	ES	1.186 (0.516–2.724)				
Week Sport Volume	≤Age	0.714 (0.358–1.424)	0.339	2.258	0.133	0.113
	>Age	1.400 (0.702–2.792)				
Annual Sport Volume	≤8 months	0.251 (0.025–2.545)	0.242	2.578	0.211	0.120
	>8 months	3.985 (0.393–40.410)				
Training Leisure Ratio	≤2:1	0.685 (0.323–1.451)	0.323	3.089	0.079	0.132
	>2:1	1.460 (0.689–3.093)				
Maturity	Pre/Early Pubertal	1.218 (0.630–2.355)	0.558	0.001	0.970	0.003
	Mid/Late Pubertal	0.821 (0.425–1.588)				

**TABLE 7:** The association of gradual onset injury with specialization, sports volume, and maturity.

Independent variable		Odds ratio (95% confidence interval)	*p*	χ 2	*p*	Phi
Level of Specialization	No ES	1.113 (0.477–2.596)	0.804	0.057	0.811	0.018
	ES	0.899 (0.385–2.096)				
Week Sport Volume	≤Age	0.957 (0.475–1.927)	0.902	0.020	0.888	0.011
	>Age	1.045 (0.519–2.104)				
Annual Sport Volume	≤8 months	0.943 (0.146–6.107)	0.951	0.002	0.968	0.003
	>8 months	1.060 (0.164–6.865)				
Training Leisure Ratio	≤2:1	1.003 (0.475–2.118)	0.994	0.000	0.997	0.000
	>2:1	0.997 (0.472–2.105)				
Maturity	Pre/Early Pubertal	1.053 (0.551–2.013)	0.875	0.035	0851	0.014
	Mid/Late Pubertal	0.949 (0.497–1.815)				

## 4 Discussion

This study aimed to: 1) investigate the training situation of Chinese elite youth football players under the new youth football player development model (SFP), exploring the category to which this model belongs; and 2) examine the associations of early specialization, sports volume, and maturity status with musculoskeletal injury in Chinese elite youth football players. The results showed that within the context of SFP, most elite football players (80.3%) followed the early specialization pathway. This study found that most injuries occurred in the lower limbs, the most common location of injury was the ankle, and the most common type of injury is an acute injury. To our knowledge, this is the first research that specifically examines the early specialization and maturity of Chinese youth players.

### 4.1 Relationship between injury and early specialization

The first contribution of this study is to identify early specialization was weakly associated with the odds of reporting at least one injury history. Early-specialized players have been found significantly to be more likely to report at least one injury in the past 12 months and more likely to report an acute injury than non-early-specialized players, which has been proved in previous studies (Jayanthi et al., 2015; Brenner et al., 2016; Post et al., 2017). Therefore, this study provides further evidence that early specialization increases the risk of injury in athletes. At present, the reasons why early specialization will increase the risk of injury to athletes can be divided into three categories: 1) Improper training load (Drew and Finch, 2016); 2) Youth players did not yet fully grow physically and were not prepared for high-intensity specialized training. Sport specialization might lead to biomechanical deterioration before maturity, which could worsen through the different phases of maturation of young players (Le Gall et al., 2007; DiCesare et al., 2019); 3) Sport specialization in adolescence limited the abilities of athletes. Instead of experiencing the varied load-adaptive stimuli of a variety of sports, they only focused on the motor skills to repeat their sport and neglected the development of other “basic” motor skills, thereby limiting the development of neuromuscular patterns of injury prevention and potentially developing exercise strategies that increase the risk of injury (Mostafavifar et al., 2013; LaPrade et al., 2016; DiCesare et al., 2019). As a result, researchers have proposed alternative pathways to early specialization, the early diversification pathway and the early engagement pathway (Baker, 2003; Côté et al., 2007; Ford et al., 2009), which aimed at increasing the participation of athletes in other sports or self-lead sports by adolescents for fun. Because some studies have supported the idea that a variety of sports participation may limit the potential risk of injury (Coté et al., 2009; Jayanthi et al., 2015). An important task of football youth training is to prevent injuries due to the high cost of injuries. Injuries may not only lead to functional impairment and reduce sports participation (Gabbe et al., 2003) but also increase the possibility of future injuries and long-term health risks (Swain et al., 2018), thereby undermining the potential benefits of sports. Athletes experiencing a greater injury burden may be due to experiencing frequent injuries early in their athletic careers (Krabak et al., 2021). Therefore, for players who choose the early specialization pathway, preventing injuries is even more important.

### 4.2 Relationship between injury and sports volume

Early specialization may lead to injuries in athletes, which is linked to sports volume (Jayanthi et al., 2015; Post et al., 2017). The sports volume indicators collected in this study were those suggested by previous studies: whether athletes train more hours per week than their age or not (Jayanthi et al., 2015; Brenner et al., 2016; Post et al., 2017), whether athletes train more than 8 months a year or not, and whether the ratio of organized training to free play exceeds 2:1 or not (DiFiori et al., 2014; LaPrade et al., 2016). Our finding showed that players who trained more hours per week than their age were more likely to report leg injuries and acute injuries, which was similar to the finding of the research of Jayanthi et al. (2015). They claimed that players with a ratio of >2:1 organized training to free play were at greater risk for serious overuse injuries. However, our study did not find any significant correlation between other sports volume indicators and injuries, which may be related to the small sample size of this study and its uneven distribution on the other two sports volume indicators, because most of the participants in this study trained more than 8 months a year and exceeded a 2:1 training-to-play ratio. However, the lack of higher injuries rate in young athletes may also be related to the changes in training load and the distribution of sports volume (Myer et al., 2016). Therefore, the planning and monitoring of athletes’ sports volume is the key to preventing injuries. For athletes who choose the early specialization pathway, how to reasonably arrange and distribute their large amount of sports volume in the macrocycle of the year and the microcycle of the week is of great importance.

### 4.3 Relationship between injuries and maturity

Early specialization may lead to injuries in athletes, which is also linked to the maturity of the player (Van Der Sluis et al., 2014). The maturity status of young players affects their structural body changes and functional growth capacity such as neuromuscular (Van Der Sluis et al., 2014; Dupré et al., 2020), thereby affecting their injury risk. This study found that players who are at pre-pubertal and early pubertal were more likely to report foot injuries. Similar to the results of this study, previous studies have also suggested that Sever’s disease often occurred in athletes during pre-pubertal and early pubertal (Price et al., 2004), and this injury was considered a growth-related injury. Overuse and changes in height, weight, and body composition were considered potential causes of this type of sports injury (Adirim and Cheng, 2003; Read et al., 2018). The findings of this study provide further support for the fact that the maturity of youth players affected their sports injuries. These maturation-related sports injuries were largely thought to be preventable (Read et al., 2018). The causes of such injuries are often controllable or intervening: Teenage players experience a height spurt during puberty, and the increased leg length results in a greater moment of inertia, which in turn increases the demands on the muscles (Adirim and Cheng, 2003). The immature musculoskeletal system of young players cannot handle repetitive biomechanical stress properly, which may result in temporary delays or regressions in sensorimotor mechanisms and motor control, furthermore injuries (Jayanthi et al., 2015). An imbalance between muscle and tendon growth may also lead to an increased risk of overuse injuries (Dupré et al., 2020). During the period of the maximum growth rate of the pubertal growth spurt, changes in joint stiffness and temporary changes in bone density may result in temporary “skeletal fragility” (DiFiori et al., 2014; Van Der Sluis et al., 2014; Swain et al., 2018). As age increases, the longer time that players get exposed, the greater the training load (exercise volume and exercise intensity) they experience, the accumulation of load and the need for physical activity in young players overlap with maturity and growth, resulting in a higher incidence of injury in more mature players (Van Der Sluis et al., 2014). Previous research has also found that male youth football players allocated most of their time to matches or outdoor training, and relatively little time to strength training, so players may not be physically prepared to meet the demands of these higher training loads (Wrigley et al., 2012). The lack of scientific injury monitoring of young football players is also an important reason for their susceptibility to injury (Faude et al., 2013). Therefore, it is particularly important to design injury prevention strategies for young players based on the above reasons.

### 4.4 Injury in Chinese School Football Programme

The goal of the Chinese School Football Programme is two-fold. The primary purpose is to popularize and expand the youth football population while improving the physical and health level of students and promoting the overall physical and mental development of students; The second purpose is to discover and cultivate talents in school football. Within the SFP context, students who are interested in football can play football at school and student players who are talented in football can have the opportunity to receive high-quality training. The sample in this study has been through multiple selections, representing the highest level of school football in their age group. The results of the study show that even in the context of school, the vast majority of elite football players still choose the early specialization pathway. In our sample, choosing an early specialization pathway increases injury risk; and different levels of exercise and player maturity also affect player injury. Therefore, school football practitioners (coaches and managers) should strengthen the monitoring of players’ sports volume and maturity status, and provide targeted protection and intervention for players who follow the early specialization pathway, to reduce their risk of injury.

#### 4.4.1 Limitations

While such data may provide valuable references for youth football practitioners, there are still limitations in this study. Firstly, although the sample of this study can represent the highest level of campus football players in this age group, the sample size is not big enough that the distribution in some indicators is uneven. Secondly, the Khamis-Roche method for predicting maturity status is based on calculations of North American white ancestry, and the average error in the age group 4.0–17.5 years is about slightly above 2 cm in boys and slightly below 2 cm in girls (Cumming et al., 2017; Cumming et al., 2018). However, the sample of this study is East Asian youth football players, considering the differences of different races, and the recruitment of youth players in specific game positions may include ideal physical characteristics such as height, which may reduce the accuracy of the method.

### 4.5 Future research direction

Future research should consider comparing players’ responses to early specialization in different talent development environments (such as academies of professional clubs and football schools, etc.), to know whether the SFP can protect against or buffer the potential risks of early specialization or not. The potential risks of early specialization are not only injuries but also psychological burnout. Therefore, future research can try to explore if the SFP can alleviate the players’ burnout which may be caused by early specialization. Research on effective interventions and protective measures is important when early specialization options are the majority choice or unavoidable.

## 5 Conclusion

Our questionnaire survey data on Chinese school football elite players show that although the development environment of athletes is based on the school background, the vast majority of school football elite players still follow the early specialization pathway. Although in the context of SFP, early specialization still increases the risk of injury, especially acute injury. A player’s maturity and sports volume can affect their injury situations, specifically, pre-pubertal and early pubertal players have a higher incidence of foot injuries; players who train more hours per week than their age have more leg injuries and acute injuries. Therefore, priority protection and intervention should be carried out for groups with a high risk of injury.

## 6 Implications for practice

This study recommends that football practitioners should understand, pay attention to and promptly communicate the potential risks and evidence of early specialization to parents and players, and create a more “appropriate” environment for the healthy development of young players in daily training and competition. For example, arrange the amount of exercise reasonably, including the frequency, duration and intensity of training, as well as adequate rest and recreational time; arrange periodized strength and conditioning (e.g., Integrative Neuromuscular Training [INT]) to prevent injuries, to help athletes make full body preparation; timely monitoring and evaluation of athletes’ health, growth and development, recovery, and response to training games, especially for young players who are in a sensitive stage of growth and development and players who have chosen an early specialization pathway.

## Data Availability

The raw data supporting the conclusion of this article will be made available by the authors, without undue reservation.
